# A thioacetamide-induced liver fibrosis model for pre-clinical studies in microminipig

**DOI:** 10.1038/s41598-023-42144-8

**Published:** 2023-09-11

**Authors:** Kotaro Nishi, Hiroshi Yagi, Mana Ohtomo, Shogo Nagata, Daisuke Udagawa, Tomonori Tsuchida, Toshinori Morisaku, Yuko Kitagawa

**Affiliations:** Department of Surgery, Keio University School of Medicine, 35, Shinano-machi, Shinjuku-ku, Tokyo Japan

**Keywords:** Zoology, Diseases, Gastroenterology, Medical research

## Abstract

Drug-induced liver fibrosis models are used in normal and immunosuppressed small animals for transplantation and regenerative medicine to improve liver fibrosis. Although large animal models are needed for pre-clinical studies, they are yet to be established owing to drug sensitivity in animal species and difficulty in setting doses. In this study, we evaluated liver fibrosis by administering thioacetamide (TA) to normal microminipig and thymectomized microminipig; 3 times for 1 week (total duration: 8 weeks). The pigs treated with TA showed elevated blood cytokine levels and a continuous liver injury at 8 weeks. RNA-seq of the liver showed increased expression of fibrosis-related genes after TA treatment. Histopathological examination showed degenerative necrosis of hepatocytes around the central vein, and revealed fibrogenesis and hepatocyte proliferation. TA treatment caused CD3-positive T cells and macrophages scattered within the hepatic lobule to congregate near the center of the lobule and increased αSMA-positive cells. Thymectomized pigs showed liver fibrosis similar to that of normal pigs, although the clinical signs tended to be milder. This model is similar to pathogenesis of liver fibrosis reported in other animal models. Therefore, it is expected to contribute to research as a drug discovery and pre-clinical transplantation models.

## Introduction

The number of patients with cirrhosis continues to increase, making it desirable to achieve the challenging goals of curative treatment and control fibrosis progression. Studies on liver fibrosis require a comprehensive understanding of the complex histological changes and clinical symptoms. Therefore, validation in animal models cannot be excluded. As most chronic liver injuries are linked to liver fibrosis, various spontaneous and induced models of liver fibrosis exist^1^. A drug-induced fibrosis model involving thioacetamide (TA) administration, which is common in rodents, is frequently used because of its similarity to the pathogenesis of fibrosis in humans^1–5^. Additionally, liver fibrosis models in immunocompromised mice have been used in transplantation studies based on a logical understanding of liver injury and fibrosis^6^. While understanding the pathogenesis of liver fibrosis in small animals continues to advance, pre-clinical research requires models that resemble humans in scale and metabolic function, such as large animals and non-human primates. TA-induced fibrosis models exist in non-human primates^7,8^. However, common marmosets and macaca fascicles are difficult to handle, and their small organs make them difficult to use in pre-clinical studies^9,10^. In addition, the most significant problem in creating models with large animals is the difficulty of dose specification owing to differences in animal species and individuals. Pigs play an important role in converting transplantation, tissue engineering, and regenerative medicine into clinical practice because of their organ similarity to humans^11–16^. Microminipigs have attracted attention in long-term transplantation and regenerative medicine research because of their large litter size and low weight fluctuations^13,17,18^. The reference range of hematological normality in microminipigs has been analyzed^19^, and their CYP450 lineage, which is typical for drug metabolism in the liver, is similar to that in humans; therefore, they are suitable as human liver disease models^20^. Recently, thymectomized, immunosuppressed microminipigs have been developed and used in transplantation research^21^. Immunomodulated animals are essential in transplantation research, thus, models of liver injury are created in mice and rats in which thymus-deficient individuals are used. Immunosuppressed animals may have altered responses to injury and fibrosis induction, and require an understanding of the pathophysiology that may be different from that of normal animal models. A model of drug-induced liver fibrosis in these microminipigs could solve current pre-clinical research issues with the potential to accelerate transplantation medicine and drug discovery research. In this study, we evaluated the biological response and liver fibrosis in normal and thymectomized microminipigs by administering thioacetamide to establish a novel model of liver fibrosis in large animals.

## Method

### Animal

Five mature female microminiature pigs, aged (17.2–26.3 months) and weight (17.6–29.2 kg) were provided by Fuji Micra Corporation for modeling purposes. Three mature female thymectomized microminiature pigs aged (15.1–21.4 months), and weight (17.2–24.0 kg) were provided by individuals whose thymus was extracted at a young age following an existing report^21^. All experiments were designed in accordance with animal ethics and welfare and accordance with the ARRIVE guidelines. All the experiments were approved by the Institutional Animal Care Committee. The experimental procedures and protocols were approved by the Animal Ethics Committee of Keio University Tokyo, Japan (approval number: A2021-055) and were performed according to the Guide for the Care and Use of Laboratory Animals (National Institutes of Health, Bethesda, MD, USA). The pigs were housed in well-spaced cages with controlled temperature and humidity and provided fresh food and water twice daily.

### Anesthesia and euthanasia protocol

Sedation for echo and surgery was performed with medetomidine (0.01–0.02 mg/kg, iv or im), midazolam (0.1–0.3 mg/kg, iv or im), butorphanol (0.1–0.3 mg/kg, iv or im) as single agents or in combination. Laparotomy was performed by endotracheal intubation after sedation and inhalation of anesthesia with 2–3% isoflurane. Pigs were euthanized for organ evaluation at the end of the experiment. The specific euthanasia procedure involved deeply anesthetizing the pigs with 5% isoflurane, exsanguinating them, and confirming complete cardiopulmonary arrest 10 min later.

### Food intake scoring and activity scoring

To assess general conditions, food intake, and activity were evaluated based on previously reported indices in pigs^22^. Food intake was evaluated as a percentage of the initial food intake and scored as 0:0%, 1:1–25%, 2:26–50%, 3:51–75%, and 4:76–100% and was performed weekly from before the experiment to the end. The activity ratings were scored as 0: supine and immobile; 1, standing up but supine most of the time; 2, moving but relatively still; and 3, moving and active. The three raters were blinded to the assessments before the start of the experiment and at 4 and 8 weeks.

### Administration protocols and evaluation methods

The route of TA administration was adapted from existing methods used in other animal species^7,8^. The starting concentration of TA administration was set at 12.5, or 25 mg/kg, and administered thrice a week. The pigs were sedated during TA administration, and TA was administered subcutaneously in the right or left axilla or thigh, avoiding the mammary gland. The administration position was always set differently from the previous position to account for stimulation by the drug. Dose concentrations were adjusted by increasing or decreasing 12.5 mg/kg while observing their general condition. If the two veterinarians judged an acute liver injury or significant deterioration of the patient’s general condition during the administration period, the drug was withdrawn 1–3 times weekly. The pigs had a central intravenous catheter (Terumo Corp., Japan) placed through the jugular vein under isoflurane anesthesia 1 week before the start of the experiment. This catheter was used as a port for blood collection and medication, and at week 4, it was replaced with a new catheter similarly. Controls were administered saline using the same technique used for TA administration.

### Blood analysis

Blood was sampled using a catheter placed in the jugular vein. Blood samples were used for complete cell counts (Celltac α, NIHON KOHDEN CORPORATION, Japan) and biochemical blood tests (Zoetis Inc, Japan). Serum was used for hyaluronic acid measurements, liver function measurements using indocyanine green (ICG) retention rate, and cytokine measurements. The blood hyaluronic acid assay was performed using subcontracted testing (SRL, Inc., Japan). The method of measuring samples for ICG retention rate analysis was based on a previously reported conventional method^23^. Briefly described, ICG dissolved in water for injection was administered intravenously by bolus from 0.5 mg/kg through the ear vein of pigs; blood was drawn 15 min after ICG administration, and the separated serum was stored at − 80 °C until measurement. Fluorescence measurements were performed at wavelengths ranging from 805 to 884 nm. Cytokine levels in the blood before and 1 week after TA administration were measured using a Porcine Cytokine Array kit (RayBiotech Life, Inc., USA). The measurements were performed according to the manufacturer’s instructions.

### Liver biopsy

Liver biopsies were performed at week 4 to evaluate fibrosis progression. The pigs were placed in the supine position under anesthesia, and the upper abdomen was disinfected. The liver was identified using ultrasound imaging, and multiple punctures were made in the liver parenchyma of the square or medial left lobe using a 14G biopsy needle (Merit Medical Inc., USA). The collected tissue fragments were fixed by permeabilization with 4% paraformaldehyde (PFA) and subjected to histopathological examination.

### Bulk RNA-seq

Total RNA was extracted from the liver using an RNeasy Plus Mini Kit (Qiagen, USA). Figure 5 shows the primer sequences (Rhelixa Inc.) used for RNA sequencing (RNA-seq) in the transcriptome study. Library construction was performed using NEBNext® Ultra™ II Directional RNA Library Prep Kit (Illumina Inc., USA), according to the manufacturer’s instructions. Sequencing was performed using an Illumina NovaSeq 6000 platform. Expression profiles were derived as read counts and fragments per kilobase of transcripts per million mapped reads for each sample, transcript, and gene. The quality of the raw paired-end sequence reads was assessed using FastQC (Version 0.11.7). The calculated quality scores were of high quality (> Q35). Low quality (< 20) bases and adapter sequences were trimmed using Trimmomatic software (Version 0.38) with the following parameters: ILLUMINACLIP 2:30:10 LEADING:20 TRAILING:20 SLIDINGWINDOW:4:15 MINLEN:36. The Benjamini–Hochberg approach was also used to identify differentially expressed genes (DEGs) using the criteria of log2 (Fold Change, FC)| & gt; 1 and adjusted *p* value (padj) < 0.05. Using ggVolcanoR, a volcano graphic was produced using FC and the padj values of the DEGs.

### Histological analysis

Tissues infiltrated in 4% PFA were thinned at 2 μm and used for histopathological analysis. Two or more expert experimental pathologists scored inflammation and fibrosis assessments of the liver tissue using hematoxylin and eosin (H&E) staining and AZAN staining (September Sapie Co., LTD, Japan). Fibrosis area assessment using Sirius red-stained samples was performed using the ImageJ software^24^. The average of the positive areas in five randomly captured fields of view was quantified. Immunohistological analyses were performed using standard methods with commercially available antibodies. Information regarding the antibodies used is provided in (Supplementary Table 1). After rinsing, the sections were mounted using DAPI and ProLong Diamond Antifade Mountant (Invitrogen, Carlsbad, CA, USA). The slides were imaged using a BZ-X810 microscope (KEYENCE Co., Japan). To evaluate the area of CD3-positive cells in the liver lobule, the percentage of positive cells in the liver lobule was calculated per area within the three divided lobule areas. Collagen hybridization peptide (CHP) staining (3Helix Inc., USA) was performed according to the manufacturer’s protocol using probes to detect remodeled collagen.

### Statistical analysis

Statistical data were analyzed using EZR, an R-based analysis software^25^. Repeated-measures analysis of variance (ANOVA) and Bonferroni test were used for weekly comparisons. Mann–Whitney U tests were used to compare parameters for group comparisons. Statistical significance was defined as a *p* value < 0.05.

## Result

### TA-treated pigs induce a decrease in general conditions with an increase in blood cytokines

**Figure 1:**
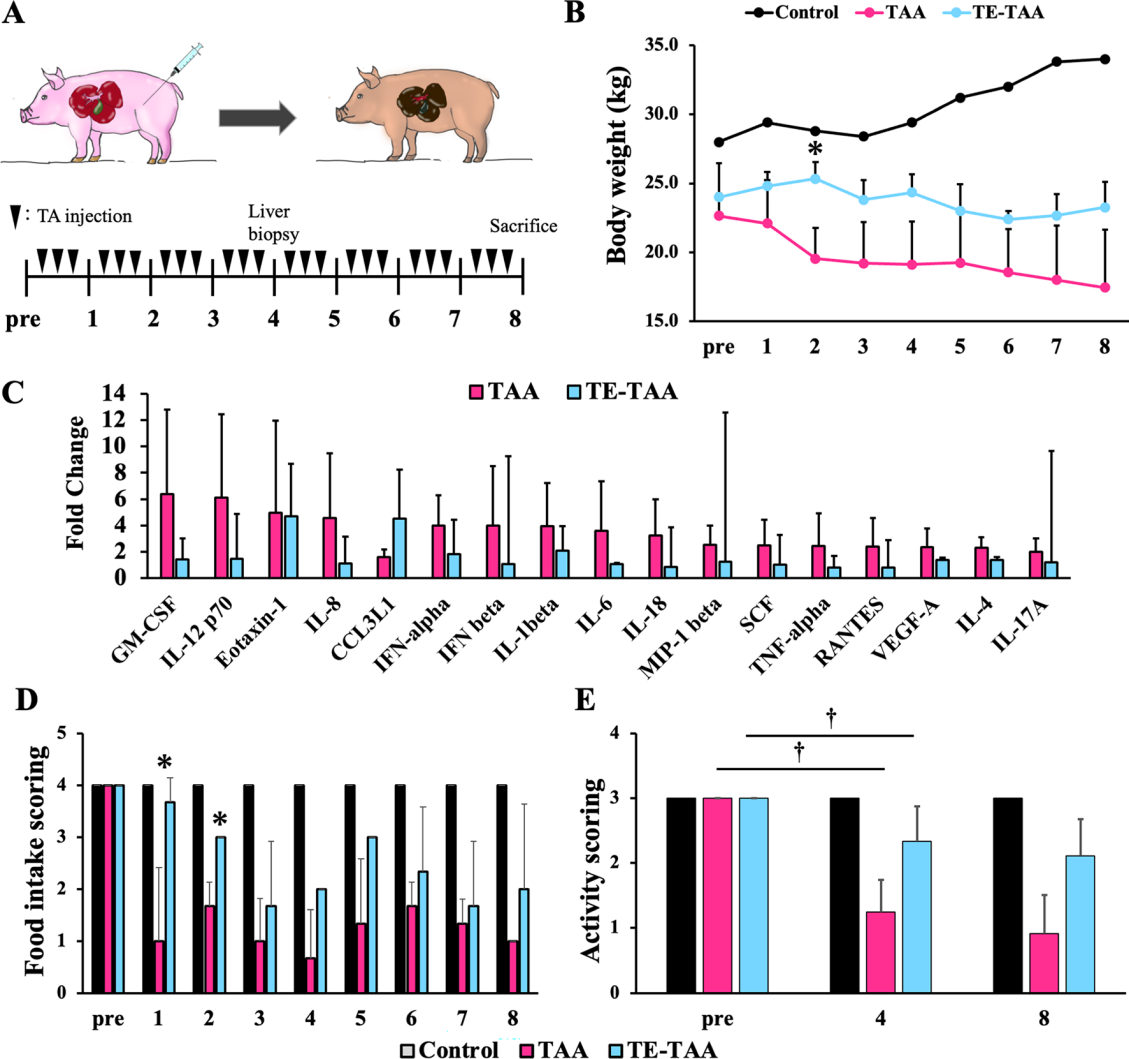
(**A**) Scheme depicting TA administration to microminipigs is shown. The TA is withdrawn according to the pig's physical condition. For more information on drug withdrawal, refer to Table 1. (**B**) Body weight changes at 8 weeks of observation. (**C**) Elevated cytokine concentrations before and 1 week after administration in TAA and TE-TAA groups were shown as fold change. Cytokines elevated more than twofold were picked up and shown in the graph. (**D**) Food intake changes during 8 weeks are shown (*: TAA vs. TE-TAA, *p* < 0.05). (**E**) Activity changes at pre, 4, and 8 weeks of the experiment are shown. (†: pre vs. 4 weeks, *p* < 0.05).

The information on all the pigs used, the total dose of TA administered, and a summary are presented in Fig. 1A and Table 1. The thymus glands of immunosuppressed pigs (TE-TAA group) were surgically removed on the day of birth. The pigs were raised under appropriate conditions before the experiment. There was no evident residual atrophic thymus at the thymectomy site in TE-TAA pigs compared to that of normal pigs, and blood thymosin levels tended to be lower (Supplemental Fig. 1). There was no significant difference in the amount of TA administered between the TAA and TE-TAA groups, and no pigs died during the observation period (Table 1). The blood test results and general condition of the thymectomized pigs were equivalent to those of the normal pigs before the start of the experiment. The pigs received subcutaneous doses of TA based on body weight, resulting in a small area of quickly healed dermatitis at the administration site. TA-treated pigs tended to lose weight and showed no significant improvement throughout the 8 weeks (Fig. 1B). Several pigs showed substantial deterioration in their overall health, resulting in vomiting and nausea, necessitating rest (Table 1).

**Table 1:** Individual information on the microminipigs is presented. Body weights measured at pretreatment and 8 weeks after treatment. For the TA withdrawal period, each withdrawal is counted as one.

	No	Sex	Age (month)	Body weight (kg)		Total injected TA volume	Total drug withdrawal (time)
				Pre	8 week		
Control	No. 1	F	26.3	28	34	–	–
TAA	No. 2	F	26.1	29.2	24.2	582 mg/kg (16,995 mg)	4
	No. 3	F	25.9	18.6	17.4	430 mg/kg (8.002 mg)	4
	No. 4	F	21.4	19.8	14.6	336 mg/kg (6,645 mg)	7
	No. 5	F	17.2	17.6	13.4	306 mg/kg (5267.5 mg)	6
TE-TAA	No. 6	F	15.1	17.2	23.8	569 mg/kg 9782.5 mg	–
	No. 7	F	19.6	24	25.2	311 mg/kg 7,467.5 mg	2
	No. 8	F	21.4	23.8	20.0	301 mg/kg 7,172.5 mg	2

After TA administration, blood cytokine levels increased several-fold within the first week compared to the pre-treatment levels. The cytokines shown in Fig. 1C are those that were more than twofold higher in cytokine concentrations in the TAA or TE-TAA groups compared to pre-treatment. There was an increase of more than twofold in inflammatory cytokines, including interleukins 1, 4, 6, 8, and 12. In addition, chemokines involved in granulocyte and macrophage recruitment and inflammation initiation were listed. Supplemental Fig. 2A shows factors with less than twofold change in cytokine concentration. In contrast, after TA administration, cytokine levels did not significantly increase in several pigs (Supplemental Fig. 2B).

**Figure 2:**
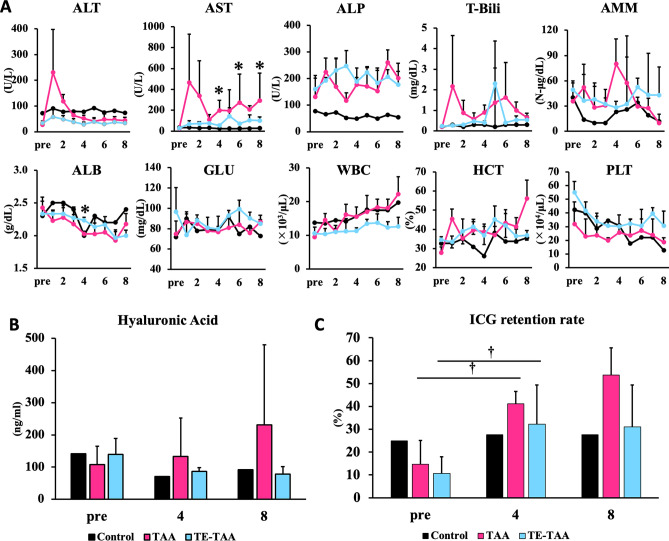
(**A**) Blood biochemical test results. (*: TAA vs TE-TAA) (**B**) Blood hyaluronic acid at pre, 4, and 8 weeks after injected TA. (**C**) ICG retention rate at pre, 4, and 8 weeks after injected TA. (†: pre vs. 4 weeks, *p* < 0.05).

Food intake and activity decreased over time in all pigs (Fig. 1D, E), and the TE-TAA group showed a milder decrease in food intake compared to that in the TAA group (Fig. 1D).

### Subcutaneously administered TA induces liver injury and impaired liver function in microminipigs

The results in Fig. 2 show acute liver injury in pigs one week after TA administration, indicated by elevated levels of liver enzymes, including ALT, AST, and ALP (Fig. 2A). Acute liver injury causes a transient elevation in most parameters. However, discontinuation of drugs results in improvement of transient increases, while AST and ALP levels continue to show elevation. In some weeks, liver enzymes were milder in TE-TAA compared to that in TAA. In addition, individuals treated with TE-TAA had a slower decrease in ALB values. Blood hyaluronic acid (HA) levels were markedly elevated in several pigs at weeks 4 and 8 (Fig. 2B). The retention rate of ICG was higher than its initial value (Control, TAA, TE-TAA: 24.9%, 14.7%, and 10.7%, respectively) at week 4 (Control, TAA, TE-TAA: 27.6%, 41.1%, 32.3%, respectively) and week 8 (Control, TAA, TE-TAA: 27.6%, 53.8%, 31.0%, respectively), confirming continued hepatic deterioration (Fig. 2C). No apparent histopathological abnormalities were found in the lungs, hearts, or kidneys at week 8 (Supplemental Fig. 3).

### Four weeks of TAA administration promotes hepatic lobular changes that can be evaluated by liver biopsy

**Figure 3:**
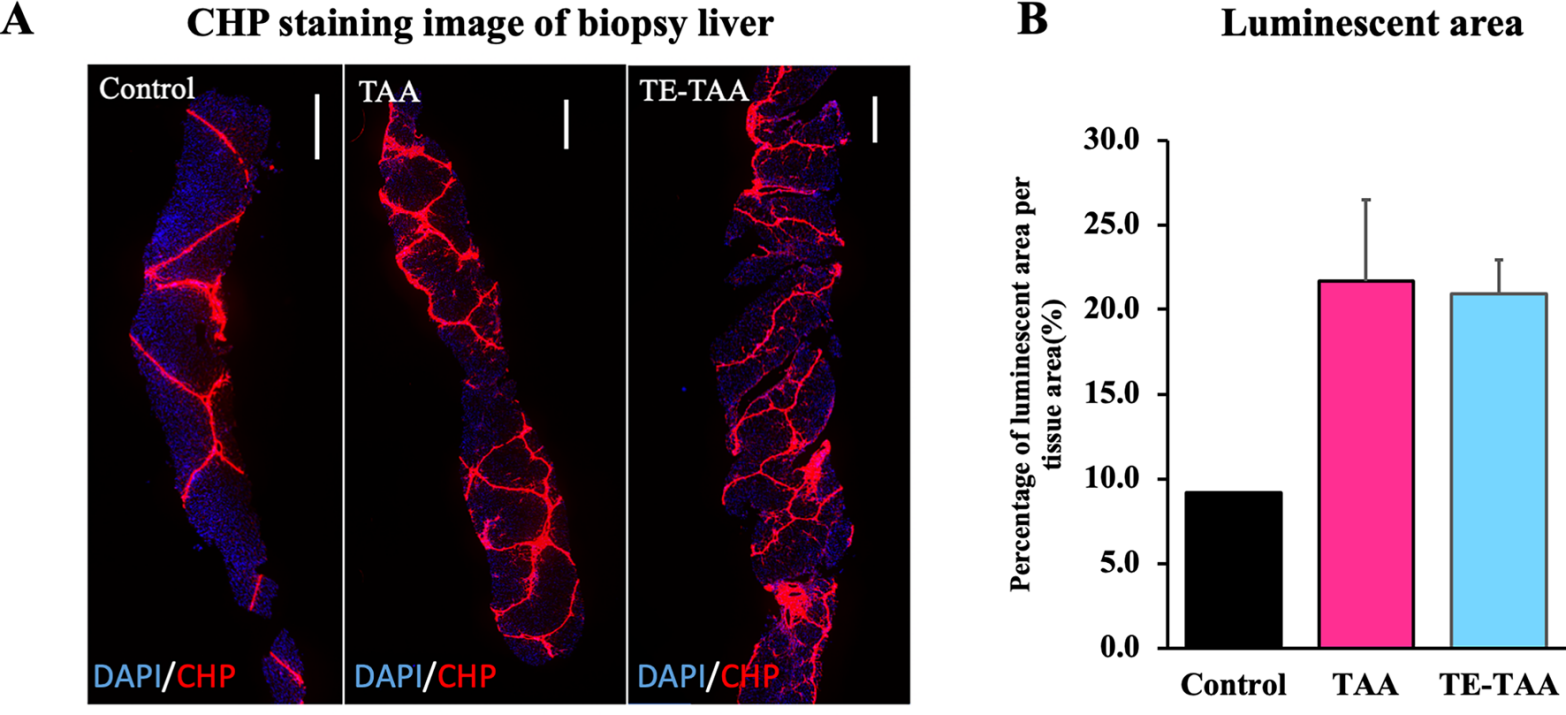
(**A**) Fluorescence-observed biopsy liver samples are shown using CHP staining. DAPI is represented in blue, and CHP in red. (**B**) The area of fluorescent regions was calculated as a percentage per tissue area. There is no difference in the percentage of TAA and TE-TAA.

All pigs underwent echo-guided liver biopsy under anesthesia at week 4 to observe for changes in the liver. TA-treated livers showed extensive hepatocellular necrosis within the hepatic lobules. In addition, CHP staining was performed to detect the collagen produced during liver injury remodeling (Fig. 3A). Studies have demonstrated that CHP probes are effective across all species and collagen types^26^. The CHP staining allowed us to stain the collagen fibers in the liver biopsy. The individuals treated with TA showed a higher CHP fluorescence area. At the same time, the control pig livers only had minimal staining of collagen surrounding the liver lobule (control vs. TAA, TE-TAA: 9.2% vs. 21.7%, 20.9%) (Fig. 3B). The livers of both TAA and TE-TAA showed a high luminescent area at CHP after 4 weeks, with no difference.

### Sustained TAA administration alters the localization of inflammatory cells in the hepatic lobules

**Figure 4:**
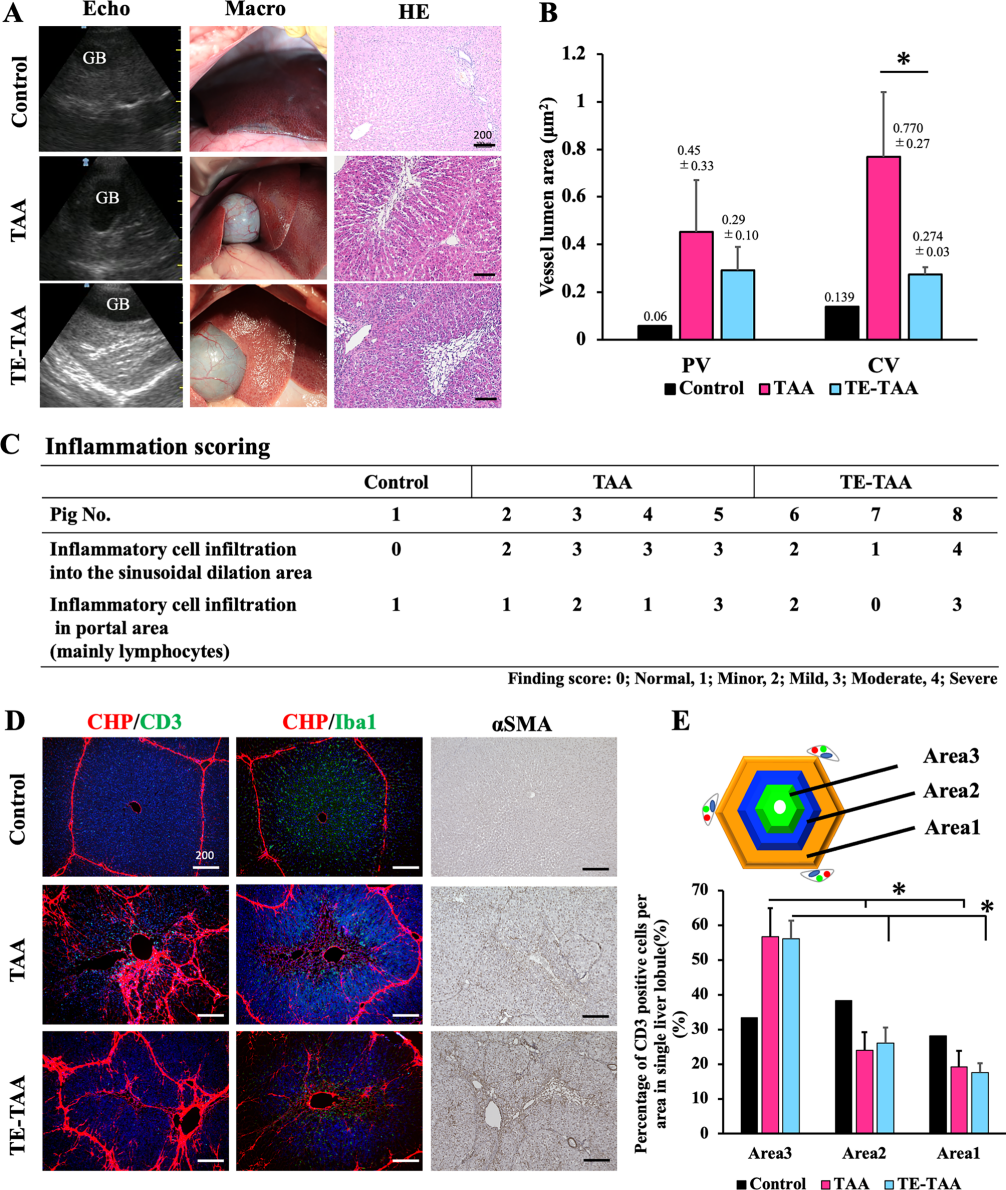
(**A**) Echographic, macro, and HE images at 8 weeks. (**B**) The area of the portal and central venous vessel lumen in the liver lobule was evaluated. Dilation of portal venules (PV) and main vein (CV) vessels compared to control vessels. (*: TAA vs TE-TAA) (**C**) Inflammation scoring of the liver tissue as assessed by an expert pathologist is shown. (**D**) Immunohistochemical staining images of CD3, Iba1, and αSMA. (**E**) Percentage of CD3 in the liver lobule is divided into three areas. Area 3 had a higher percentage of CD3-positive cells than the other areas. (*: Area 3 vs Area 1 or Area 2).

TA-treated livers in both groups showed numerous ridges consisting of hepatic lobular areas on the capsular surface, which were not observed in the normal livers (Fig. 4A). Echocardiographic findings revealed a vascular wall with high echogenicity. H&E staining revealed lobular central hepatocellular necrosis and localization of inflammatory cells in the hepatic lobules. The hepatic lobule's central vein and portal venules were dilated (Fig. 4B) and eosinophilically stained; unoriented fibrils were observed around the vessels. An experimental pathologist concluded inflammatory cell migration due to TA administration by evaluating inflammation in the sinusoidal and portal regions (Fig. 4C). The localization of T lymphocytes, macrophages, and αSMA-positive cells in hepatic lobules was investigated to understand the histological effects of TA on the pathophysiology of hepatic fibrosis (Fig. 4D). CD3-positive lymphocytes and Iba1-positive macrophages were observed in the liver lobules of the Control, TAA, and TE-TAA groups. The livers of control pigs showed scattered lymphocytes and macrophages in the hepatic lobules. Livers administered with TA had lymphocytes clustered in the area nearest to the center of the lobule (Fig. 4E). In the control livers, few αSMA-positive cells were observed; however, many αSMA-positive cells were observed in the liver administered TA. The livers of the control group were macroscopically smooth and did not differ from echography or histological findings in commonly used healthy pigs (Supplemental Fig. 4).

### Eight weeks of TAA administration to microminipigs simulate the pathophysiology of fibrosis with hepatocyte proliferation

**Figure 5:**
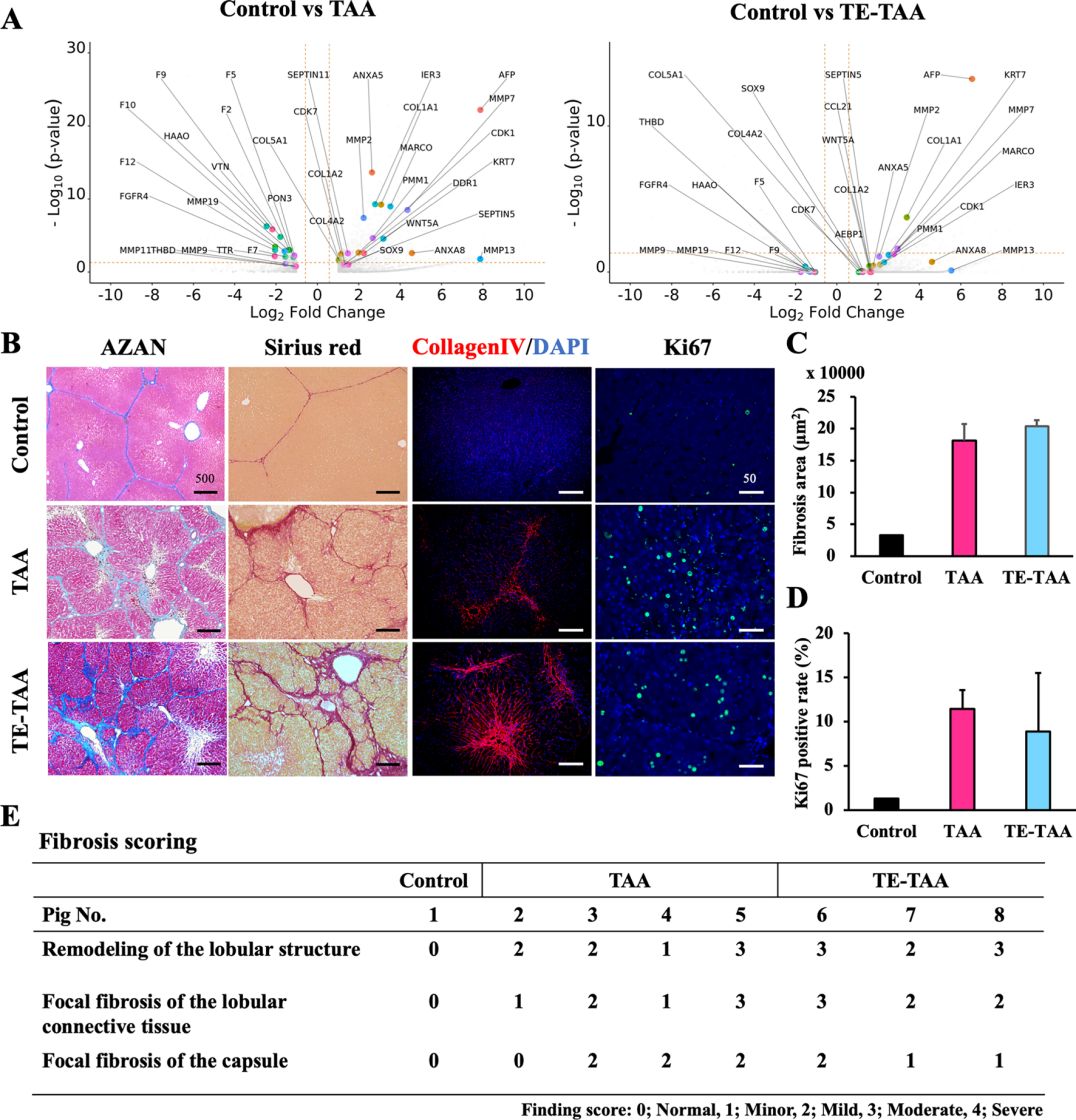
Volcano plots of the liver RNA-seq expression analysis results of TAA and TE-TAA compared to that of the control. DEGs used the criteria of |log2 (Fold Change, FC)| &gt; 1 and adjusted *p* value (padj) < 0.05. (**B**) Staining images of AZAN, Sirius red, Collagen IV, and Ki67. (**C**) Fibrosis area calculated from Sirius red staining positive area. (**D**) Ki67-positive cell rate per 100 hepatocytes expressed as a percentage. (**E**) Liver fibrosis scoring by pathology experts is shown.

The RNA-seq analysis of the excised liver tissue is shown in Fig. 5A, and compared to the control, gene expression in fibrosis pathologies, such as COL1, MMP, and AFP, increased in both the TAA and TE-TAA groups. In addition, genes encoding coagulation factors involved in liver function were downregulated. The hepatic lobular architecture was reconstructed, and increased fibrosis was observed from the lobular center to the Gleason region on AZAN and Sirius red staining (Fig. 5B). The injured livers contained many liver cells of variable sizes that stained positive for Ki67. Ki67-positive cells were scarce in control hepatocytes and were highly enriched in hepatocytes from injured livers (control vs. TAA, TE-TAA:1.3% vs. 11.5%, 8.9%). No differences between the TAA and TE-TAA groups were observed in fibrotic areas or Ki67 positive cells (Fig. 5C, D). Histopathological analysis scoring for liver lobular structure remodeling, cross-linking fibrosis, and capsular fibrosis clearly showed differences from control (Fig. 5E). In all pigs, fibrous stroma was confirmed to constitute a bridge between the central vein and the portal venules. The control livers were smooth and did not differ from the normal pig livers on echography and histological analyses.

## Discussion

Translation-based research is being conducted in mechanistic analysis and drug discovery for treating liver fibrosis. However, there are frequent problems with poor extrapolation owing to the scale and dosage of the animals. Additionally, metabolic function and histological morphology differ among animal species, and there is a need to establish pathological models that correspond to animal species. We had previously established a model of liver injury in pigs by partial hepatectomy and retrorsine administration^22^. The ability of retrorsine to block the cell cycle allows hepatocytes to arrest regenerative changes and induces continuous hepatic injury. However, the liver injury caused by partial hepatectomy plus retrorsine administration was severe in pigs, and long-term observation was not achieved. It is necessary to understand, in detail, the biological responses and histological changes induced by drug administration to establish a model of drug-induced liver fibrosis in large animals as a pre-clinical model. This process will enable comparing fibrosis models in small and large animals and accelerate pre-clinical research considering animal ethics.

Although only one normal pig was used in this study, we could assemble the experiment with the minimum number of animals required. This is because the control liver in this study showed no differences from the normal livers used in previous studies. The control liver showed no evident differences from most normal pigs with regards to liver lobular structure or cellular localization, even after 8 weeks of the placebo procedure (Supplemental Fig. 4). Moreover, the fact that the blood properties of the controls do not deviate from the normative values of normal pigs also provided certainty to the analysis results in one animal. This study found that the TA dose setting could induce fibrosis in pigs without causing death. There were species and individual differences in previously reported models of drug-induced liver fibrosis; therefore, the starting doses were inconsistent^1,4,9,10^. The dosage for continued TA administration should also be adjusted according to the individual's general condition. Accordingly, the starting dose of TA for microminipigs was determined to be 12.5–25 mg/kg, a concentration of 1/8–1/4 of the 100 mg/kg frequently reported in rodents^1,4^. One basis for dose setting was each animal species' liver weight-to-body weight ratio. The liver weight-to-body weight ratios for mice and mice were approximately 5–7%, for monkeys 2–2.5%, and for pigs 2.4%^27–30^. It should be considered that dose setting by body weight conversion is not necessarily proportional to metabolic capacity and therefore does not produce drug or toxic effects^31^. Indeed, TA doses of 100–200 mg/kg have been associated with earlier mortalities in rodents, whereas some individuals die early in monkeys^8^. Indeed, TA doses of 100–200 mg/kg have been associated with earlier mortalities in rodents, whereas some individuals die early in monkeys^8^. Furthermore, CYPs, which are enzymes affecting TA metabolism, are diverse among the species and sexes of pigs^32^. Therefore, we predicted that doses similar to those used in rodents would underestimate the toxic dose, resulting in higher mortality rates. In addition, intraperitoneal administration is one of the causes of increased mortality, and modifying the route of administration may be a solution. Indeed, the subcutaneous administration of TA induces liver fibrosis in non-human primates^7,8^. According to our study, it is recommended that the method of TA administration to lower acute mortality in microminipigs should be used subcutaneously at a starting dose of 12.5 ~ 25 mg/kg or less.

In many animals, prolonged hepatotoxicity following TA administration is partly caused by the expression of toxicity. TA is metabolized by CYP2E1 to S-oxide (TASO), which covalently binds to liver macromolecules and causes cytotoxicity^33^. In addition, the reduction of TASO to TA occurs simultaneously in the liver, resulting in long-term toxicity retention and accumulation^33^. In this study, the blood levels of TA and TASO were not evaluated; however, acute liver injury with elevated cytokine levels in the blood and a rapid decrease in the general condition was observed in the first week after TA administration. Thus, there was a need to avoid multiple TA administrations. The death of cells surrounding the liver induces the production of chemokines and infiltration of inflammatory cells^3,34,35^. In rodents, levels of chemokines, such as IL-1β,6,8, are elevated after TA administration. This leads to increased neutrophils and monocytes and a worsening systemic condition^3^. In microminipigs, capturing similar phenomena and identifying acute biological responses to TA administration after three doses in one week were possible.

The progression of liver fibrosis from liver injury was defined as hematological and histopathological changes after the continuous administration of TA to microminipigs. Generally, HA and collagen 4 in the blood are biomarkers to observe the development of liver fibrosis^36,37^. Activated hepatic stellate cells (HSC) express hyaluronan synthase (HAS2)^38^, which is involved in the accumulation of HA in the liver. HAS2 and HA further promote HSC activation and fibrosis^38,39^ and are detected in the blood of animals with advanced liver fibrosis^38^. In this study, HA levels were elevated in some individuals in the TAA group. We conclude that HA could be an indicator of liver fibrosis in microminipigs, as HA tended to increase temporally and was linked to an increase in the ICG retention rate. However, collagen 4 could not be confirmed elevated in the blood, even though it was detected histologically. It is known that type 1 collagen is more variable than type 4 collagen in the ECM ratio in the fibrotic pathology of the liver^37^. These results could be interpreted as COL1-predominant fibrosis since there was an apparent increase in COL1 over COL4 gene expression. The TAA and TE-TAA groups showed elevated expression of fibrosis-related factors, such as AFP and MMP2,7, following TA administration in rats^40^. In addition, the expression of some fibrosis progression signature (FPS) genes, which have attracted attention as predictors of fibrosis, tends to increase^41^. These results suggest that continued TA administration may further enhance fibrosis and that the FPS gene may be used to predict liver fibrosis in microminipigs, enhancing biopsy-based diagnosis. The liver showed gross and histopathological evidence of fibrosis. Echo evaluation in the microminipigs showed a highly echogenic image of blood vessels, although ultrasound transmission was low because of thick skin. This result is consistent with the histological findings of perivascular fibrosis and vasodilation. Meanwhile, the liver parenchyma could not be recognized as an evident difference on ultrasound. Although there are few reports on pigs, ultrasound elastography has been shown to have the potential to distinguish low fibrosis stages, as represented by METAVIR^35,42^. Elastographic evaluation, which could not be performed in this study, has the potential to assess fibrosis while reducing biopsy damage and pig burden.

The behavior of immune cells during liver injury is one factor that influences fibrosis and anti-fibrosis. The complex behavior of various immune cell types in the pathogenesis of liver fibrosis has been validated in animal models. However, there are fewer reports on the cellular behavior of drug-induced liver injury in larger animal models of liver fibrosis than in rodent models. Hepatotoxic substances damage both zone 1 and zone 3 hepatocytes within the liver lobules in rodent livers^43,44^. Hepatic damage from TA has been reported, with zone 1 damage being more pronounced than that from other hepatotoxic substances. In contrast, the microminipigs were severely damaged in zone 3, and some liver lobules had unrecognizable central veins. This subtle difference may depend on the animal species. Rat and human livers have a higher expression of CYP450, which primarily metabolizes TA, in the pericentric venous region^45,46^. As mentioned above, the CYPs expressed vary with the species and sex of the pig. In addition, compensatory liver regeneration induced by TA differed from that induced by liver resection, in which hepatocytes adjacent to the necrotic zone underwent cell proliferation^47^. This may result in a relatively low assessment of zone 1 damage due to severe damage in areas close to the zone center. In addition, zone 3 was infiltrated by numerous lymphocytes and macrophages. The immune cells, such as T and Kupffer cells, circulate or scout the liver sinusoids and parenchyma^48^. Liver injury causes resident or blood cells to exhibit increased inflammatory and anti-inflammatory effects. Macrophages are replenished by inflammatory chemokines, such as MCP-1, which replenish blood monocytes and differentiate into monocyte-derived macrophages, increasing the macrophage pool in the liver^49,50^. Furthermore, in the TA fibrosis model in rats, the phenotype of macrophages changes during the advanced stages of pathogenesis^50^. From the results of our research, we predict that this phenomenon could also occur in microminipigs. There are substantial species-specific differences in the NK cells between humans and mice; however, pigs have NK populations that are similar to that of humans^51^. Therefore, further analysis of immune cells using TA in pigs could act as a human liver immunotherapy model. However, we could not assess a subset of these immune cells in detail as their inflammatory stage was unclear. For further analysis, there is a need to understand the species-specific inflammatory reactivity of the microminipigs. This is equally applicable to the study on thymectomized pigs. This study did not analyze the immune cells from thymectomized microminipigs in detail. However, the thymus was removed, and the liver showed evident fibrosis. Interestingly, the TE-TAA group tended to have a milder general condition and milder blood test results than the TAA group. Thymectomized microminipigs exhibit a low lymphocyte mitogen response^21^. Similarly, DiGeorge syndrome, a congenital thymic hypoplasia in humans, is characterized by decreased T cell numbers and function^52,53^. The induction of liver fibrosis in nude mice by TA treatment resulted in mild fibrinogen deposition after 5–8 weeks^54^. Contrastingly, the degree of inflammation and liver injury has not been completely elucidated. No direct evidence in this study can link immunosuppression to reduced liver injury. However, the finding that fibrosis could be induced in thymectomized pigs, as in normal pigs, suggests that thymic immunity is not significantly involved in fibrogenesis and that the effect of cytokines on the systemic status may be important for modeling.

This study has several limitations. First, we could not clarify the liver histological changes in the TAA and TE-TAA groups in detail because the main objective was to evaluate liver fibrosis caused by TA in a minimal number of animals. Fibrosis was induced in both groups; however, the influence of immune cells on pathological conditions related to fibrosis and inflammation remains unclear. How long fibrosis is maintained after TA administration is withdrawn is also unclear. This issue has frequently been discussed, and fibrosis is maintained for 4–8 weeks after the discontinuation of TA administration^43,55^ in rats. However, the duration may vary among animal species owing to protease effects; therefore, the status of the liver after withdrawal of TA administration should be investigated in the future.

In conclusion, this study establishes a protocol for TA-induced liver fibrosis in microminipigs. This 8-week protocol is considered an early establishment by several methods for animal fibrosis models^56^. Furthermore, the acute and chronic phases after injecting TA were partially elucidated in a large animal model for the first time. This model is expected to contribute to pre-clinical research requiring large animals for cell transplantation or drug discovery.

### Supplementary Information

Supplementary Information.

## Data Availability

All raw data sets are deposited in DDBJ Sequence Read Archive (DRA) with bioproject accession number PRJDB16132. The original contributions presented in this study are included in the article/Supplementary Material. Further inquiries can be directed at the corresponding authors.
