# Supplementary material for: A thioacetamide-induced liver fibrosis model for pre-clinical studies in microminipig
Source: Sci Rep. 2023 Sep 11;13:14996. doi: 10.1038/s41598-023-42144-8 (PMC10495379; doi:10.1038/s41598-023-42144-8)
Supplement: Supplementary file 1 — Supplementary Information. [file 41598_2023_42144_MOESM1_ESM.pptx]

## Slide 1
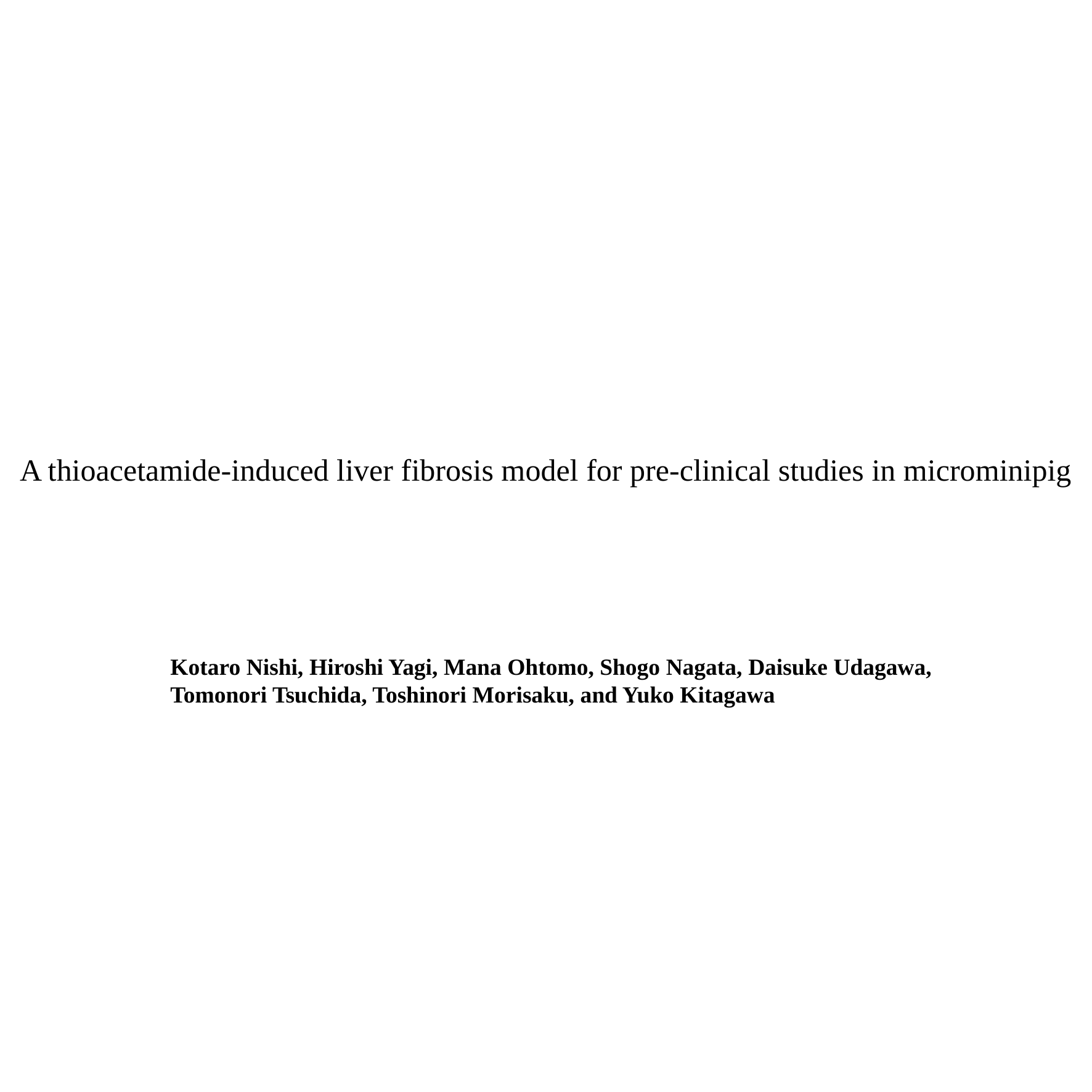

A thioacetamide-induced liver fibrosis model for pre-clinical studies in microminipig
Kotaro Nishi, Hiroshi Yagi, Mana Ohtomo, Shogo Nagata, Daisuke Udagawa, Tomonori Tsuchida, Toshinori Morisaku, and Yuko Kitagawa

## Slide 2
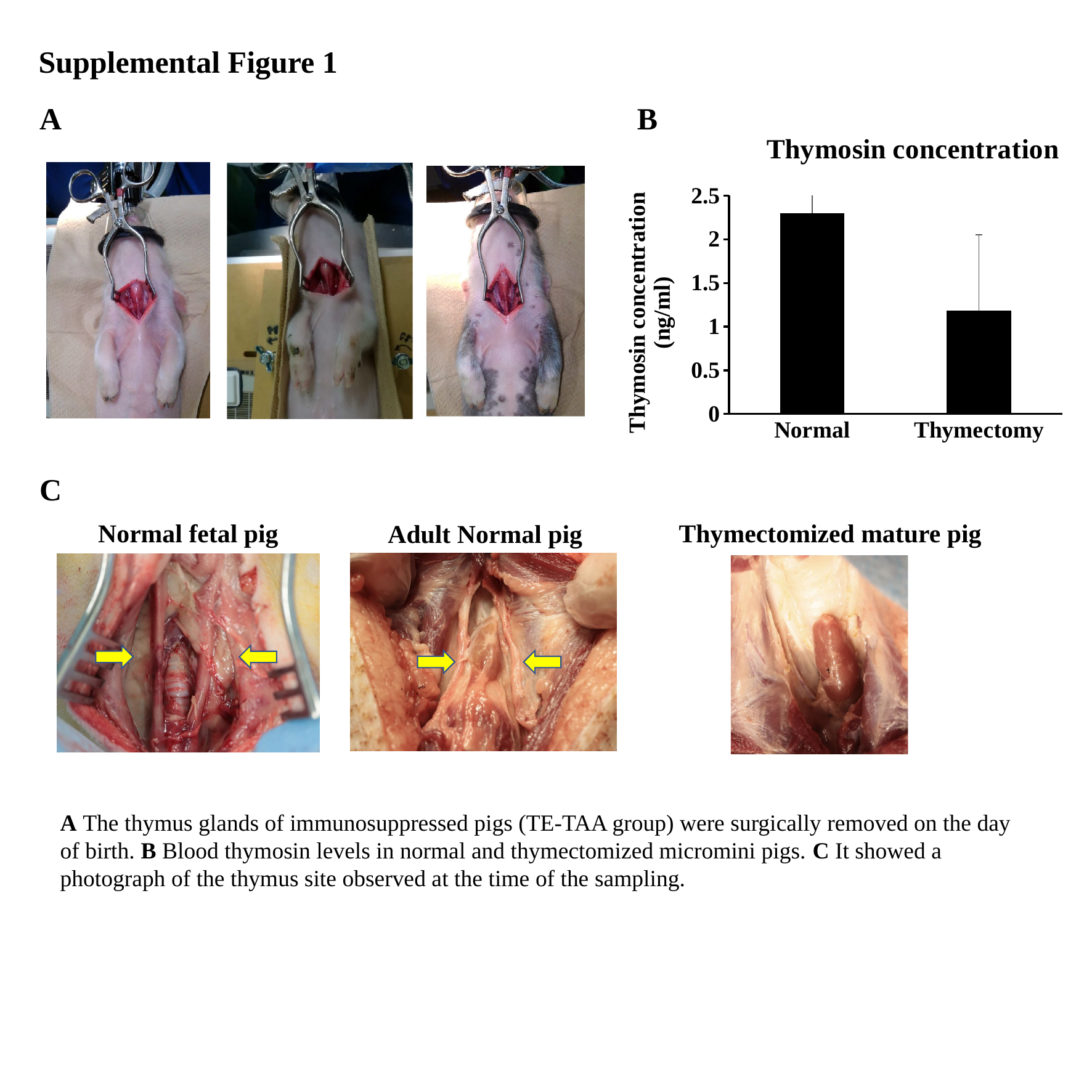

Supplemental Figure 1
A
B
### Chart: Thymosin concentration
| Category | |
|---|---|
| Normal | 2.298000015992385 |
| Thymectomy | 1.1820924303769473 |
C
Thymectomized mature pig
Normal fetal pig
Adult Normal pig
A The thymus glands of immunosuppressed pigs (TE-TAA group) were surgically removed on the day of birth. B Blood thymosin levels in normal and thymectomized micromini pigs. C It showed a photograph of the thymus site observed at the time of the sampling.

## Slide 3
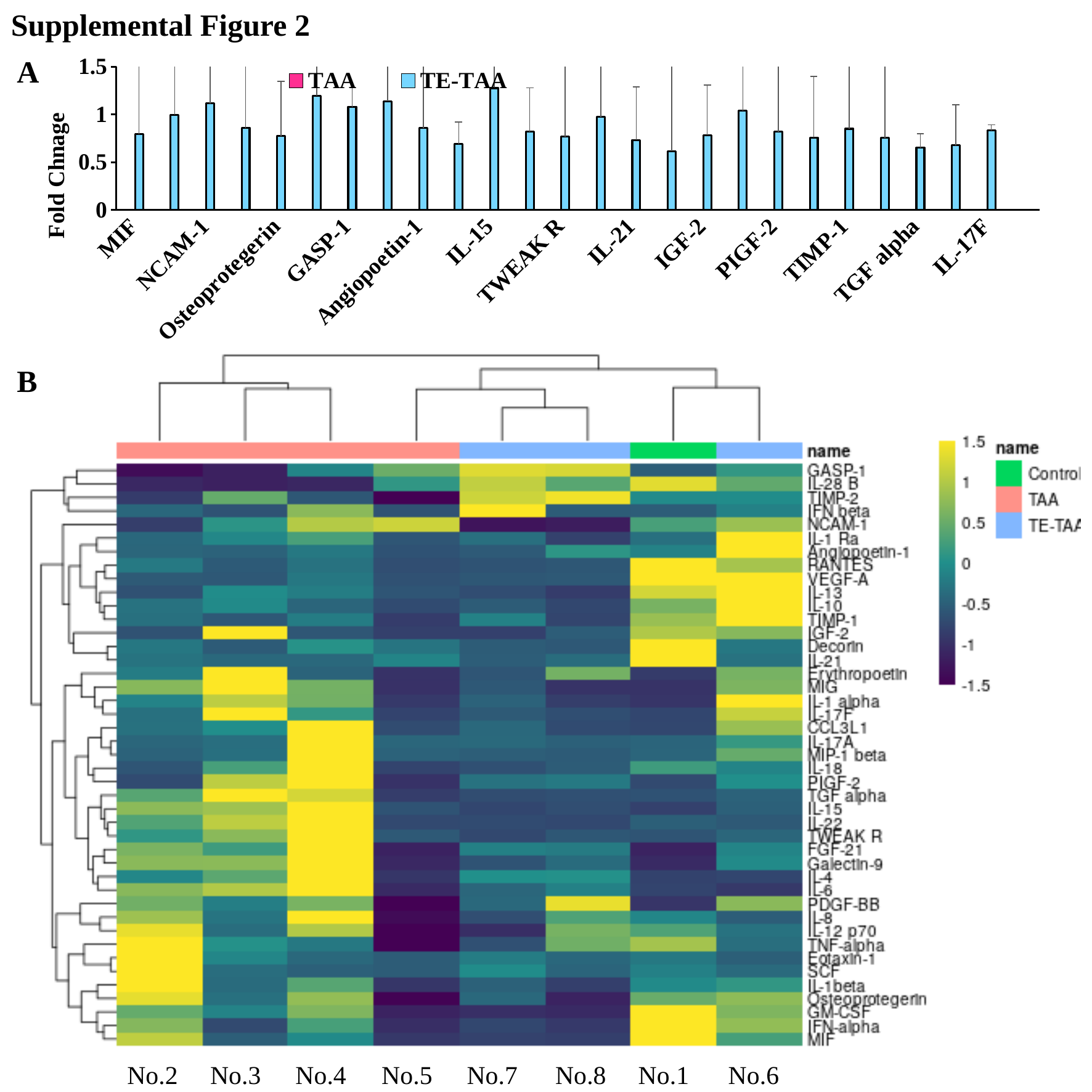

Supplemental Figure 2
A
### Chart
| Category | TAA | TE-TAA |
|---|---|---|
| MIF | 1.9543566848766147 | 0.7932985972400866 |
| FGF-21 | 1.6615088747351403 | 0.9949001059661036 |
| NCAM-1 | 1.6224629037999907 | 1.1155329303674713 |
| Decorin | 1.464747354418736 | 0.8572084928623753 |
| Osteoprotegerin | 1.4627811245898221 | 0.7734414682044981 |
| IL-10 | 1.3915977886568556 | 1.1960876294083995 |
| GASP-1 | 1.1446305070671963 | 1.0776932282886522 |
| Galectin-9 | 1.1406957378217673 | 1.1375590708592784 |
| Angiopoetin-1 | 1.1351280693845773 | 0.8583649781234766 |
| TIMP-2 | 1.123288068336185 | 0.6903736271356513 |
| IL-15 | 1.093258245922258 | 1.2741490721745306 |
| PDGF-BB | 1.0436790626606556 | 0.8190128565991275 |
| TWEAK R | 0.9564835324853422 | 0.7659726475102998 |
| MIG | 0.9555047019558879 | 0.971748290275196 |
| IL-21 | 0.9213258412374208 | 0.7312909784454029 |
| Erythropoetin | 0.9136933115425817 | 0.6133342166928285 |
| IGF-2 | 0.8836100618735905 | 0.7831392138206608 |
| IL-1 Ra | 0.8744561099806809 | 1.037246635600353 |
| PIGF-2 | 0.8740548094160691 | 0.8190934535525054 |
| IL-1 alpha | 0.8361747350501844 | 0.7558339735094123 |
| TIMP-1 | 0.8044737698611245 | 0.8494148163705187 |
| IL-22 | 0.7899495405807595 | 0.7530527864158229 |
| TGF alpha | 0.758110922713399 | 0.6499840271199386 |
| IL-13 | 0.756141942524698 | 0.6774915495599294 |
| IL-17F | 0.7320419855644938 | 0.8319639092077292 |
B
No.2
No.3
No.4
No.5
No.7
No.8
No.1
No.6

## Slide 4
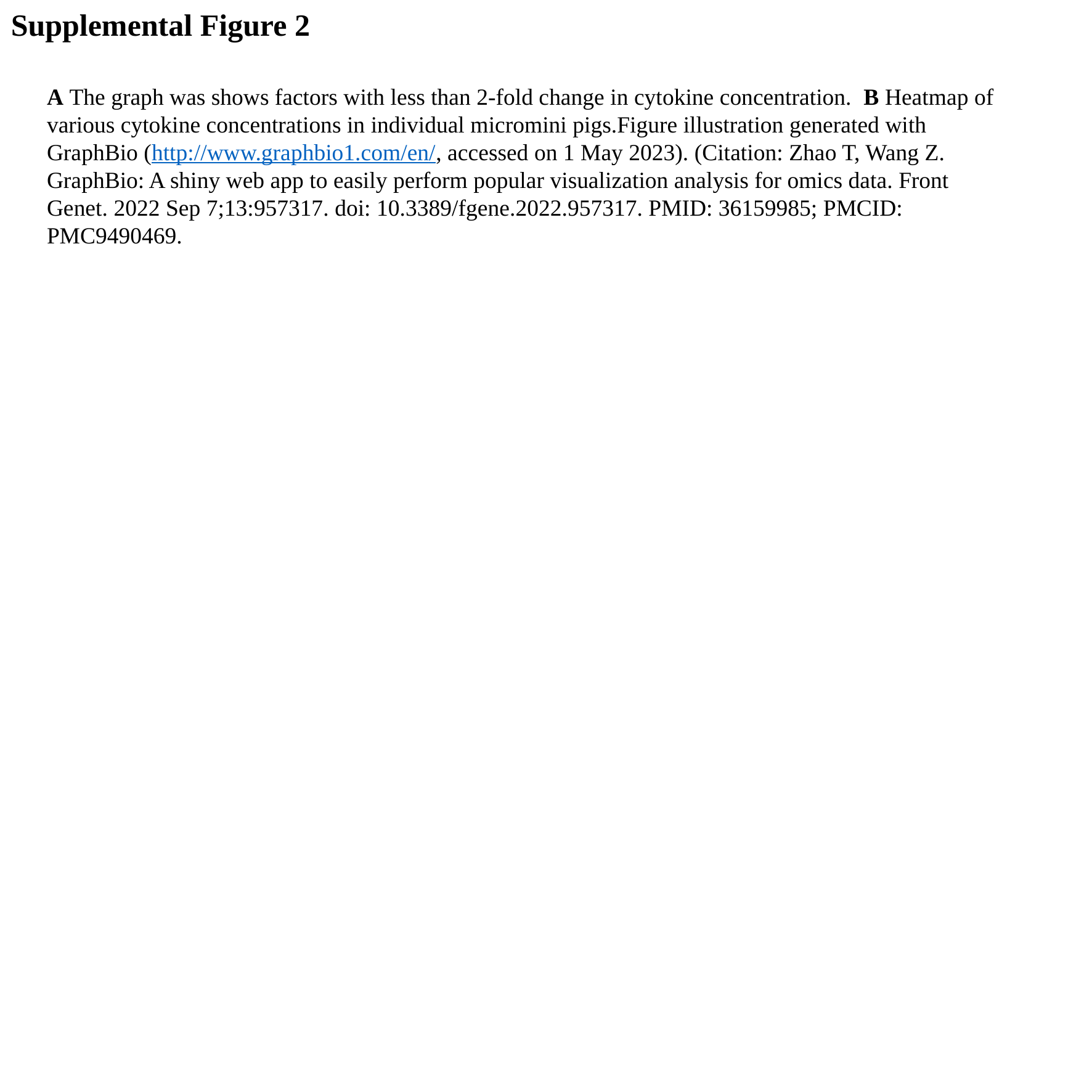

Supplemental Figure 2
A The graph was shows factors with less than 2-fold change in cytokine concentration. B Heatmap of various cytokine concentrations in individual micromini pigs.Figure illustration generated with GraphBio (http://www.graphbio1.com/en/, accessed on 1 May 2023). (Citation: Zhao T, Wang Z. GraphBio: A shiny web app to easily perform popular visualization analysis for omics data. Front Genet. 2022 Sep 7;13:957317. doi: 10.3389/fgene.2022.957317. PMID: 36159985; PMCID: PMC9490469.

## Slide 5
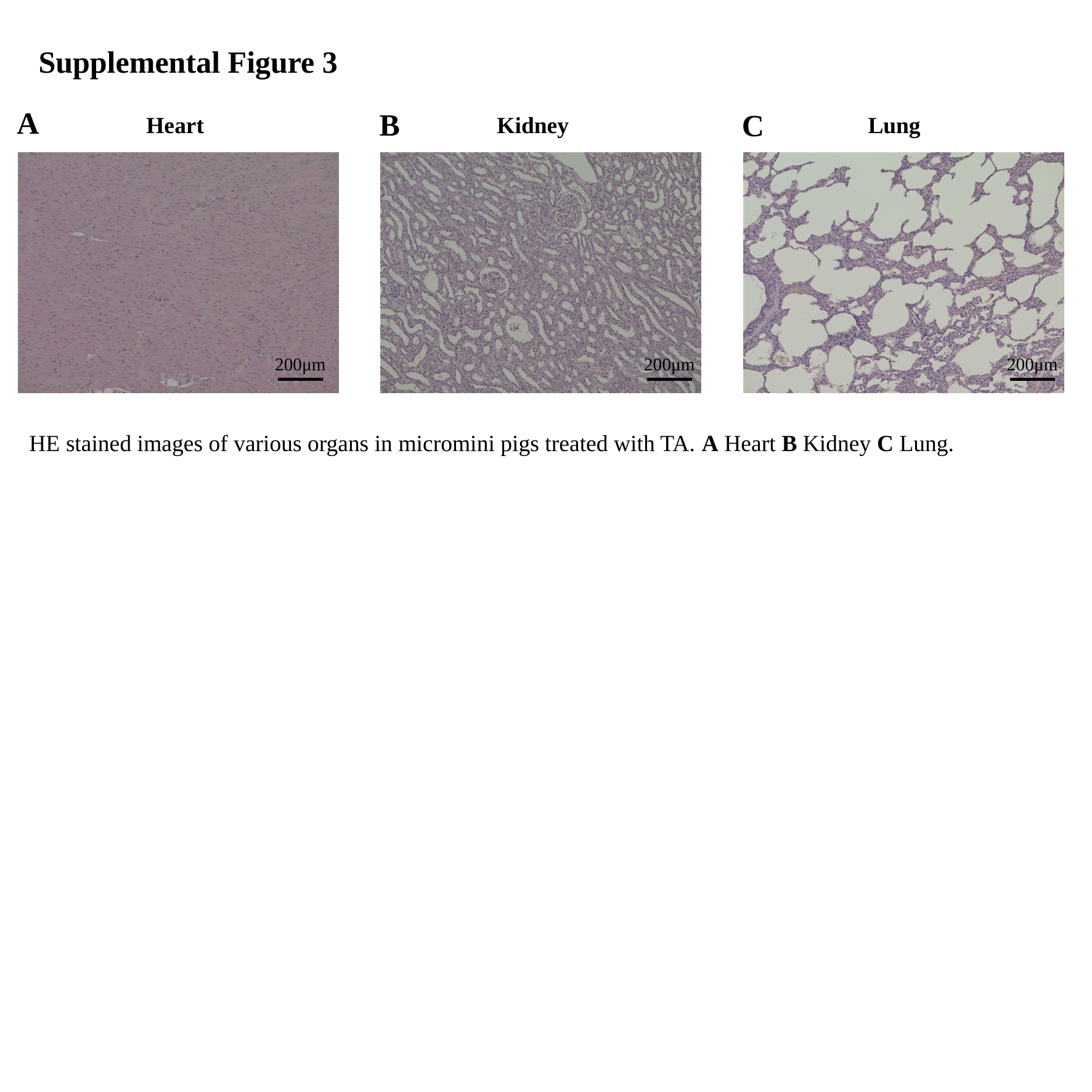

Supplemental Figure 3
A
B
C
Heart
Kidney
Lung
200μm
200μm
200μm
HE stained images of various organs in micromini pigs treated with TA. A Heart B Kidney C Lung.

## Slide 6
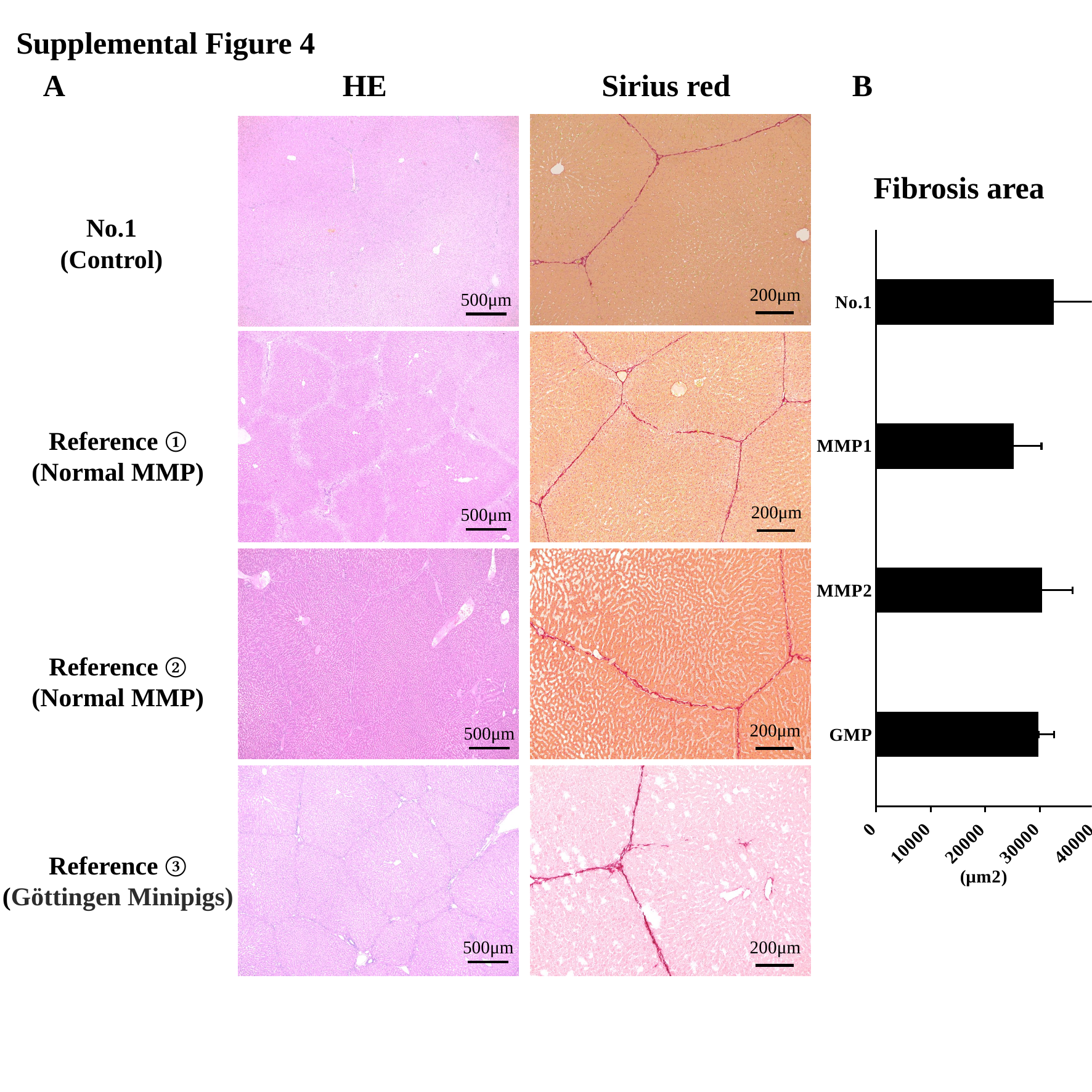

Supplemental Figure 4
A
B
HE
Sirius red
Fibrosis area
No.1
(Control)
### Chart
| Category | |
|---|---|
| GMP | 29747.2484 |
| MMP2 | 30443.821999999996 |
| MMP1 | 25204.6814 |
| No.1 | 32545.573199999995 |200μm
500μm
Reference ①
(Normal MMP)
200μm
500μm
Reference ②
(Normal MMP)
200μm
500μm
Reference ③
(Göttingen Minipigs)
200μm
500μm

## Slide 7
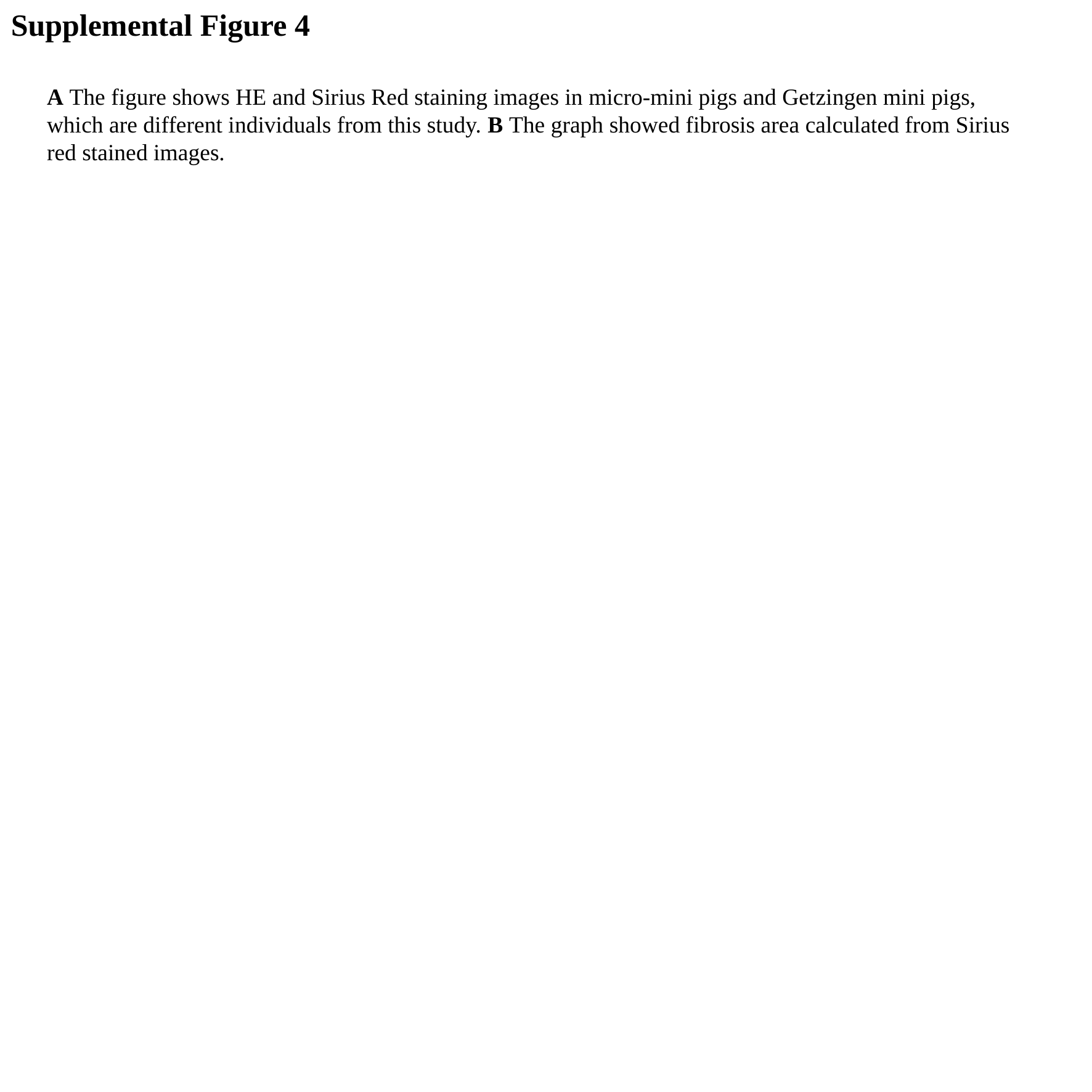

Supplemental Figure 4
A The figure shows HE and Sirius Red staining images in micro-mini pigs and Getzingen mini pigs, which are different individuals from this study. B The graph showed fibrosis area calculated from Sirius red stained images.

## Slide 8
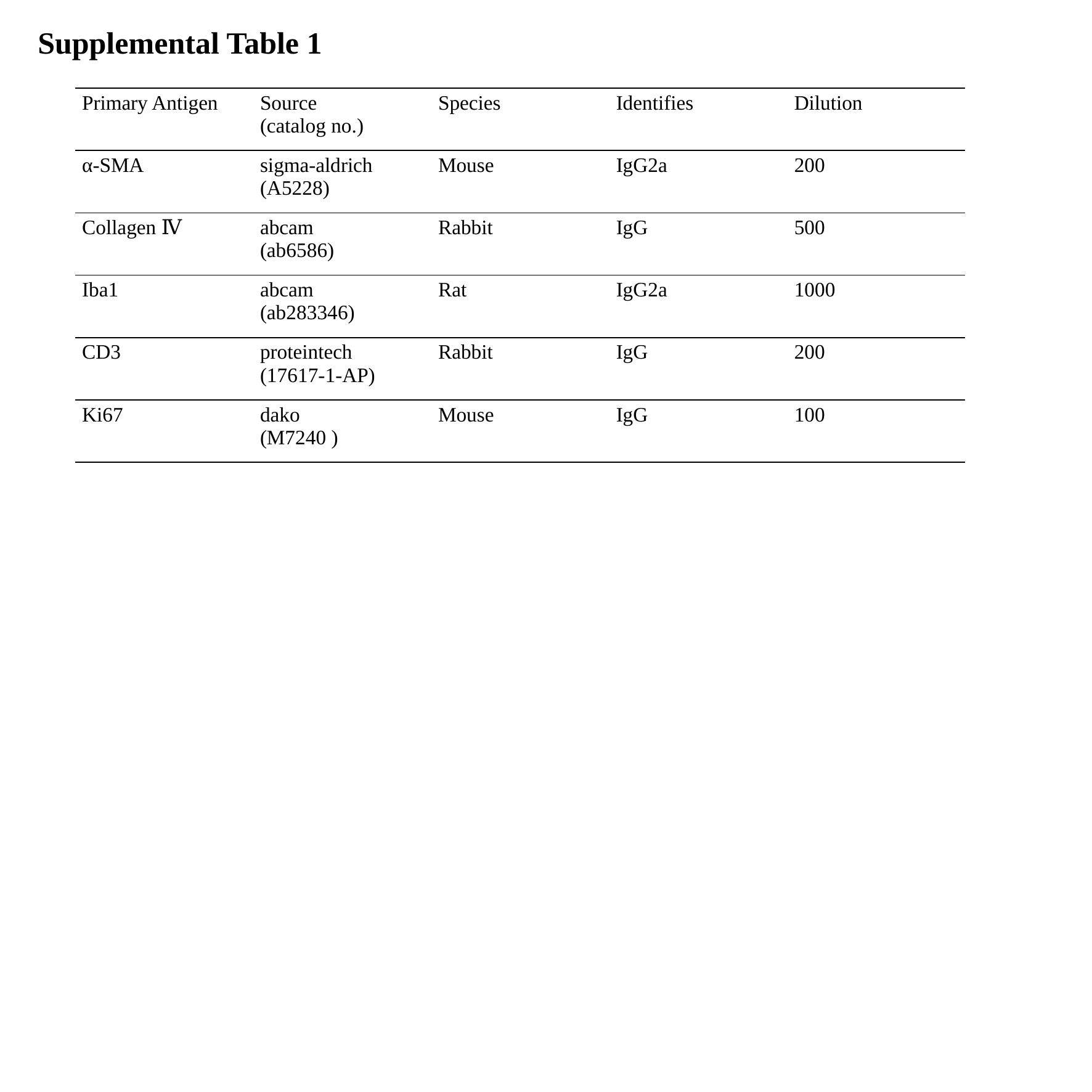

Supplemental Table 1
| Primary Antigen | Source (catalog no.) | Species | Identifies | Dilution |
| --- | --- | --- | --- | --- |
| α-SMA | sigma-aldrich (A5228) | Mouse | IgG2a | 200 |
| Collagen Ⅳ | abcam (ab6586) | Rabbit | IgG | 500 |
| Iba1 | abcam (ab283346) | Rat | IgG2a | 1000 |
| CD3 | proteintech (17617-1-AP) | Rabbit | IgG | 200 |
| Ki67 | dako (M7240 ) | Mouse | IgG | 100 |
